# Parkinson Disease and Orthostatic Hypotension in the Elderly: Recognition and Management of Risk Factors for Falls

**DOI:** 10.14336/AD.2019.0805

**Published:** 2019-08-05

**Authors:** Peter A LeWitt, Steve Kymes, Robert A Hauser

**Affiliations:** ^1^Henry Ford Hospital and Wayne State University School of Medicine, West Bloomfield, MI 48322, USA; ^2^Lundbeck, Deerfield, IL 60015, USA; ^3^University of South Florida Parkinson’s Disease and Movement Disorders Center, Parkinson Foundation Center of Excellence, Tampa, FL 33613, USA

**Keywords:** elderly, falls, neurodegeneration, neurogenic orthostatic hypotension, Parkinson disease, treatment

## Abstract

Parkinson disease (PD) is often associated with postural instability and gait dysfunction that can increase the risk for falls and associated consequences, including injuries, increased burden on healthcare resources, and reduced quality of life. Patients with PD have nearly twice the risk for falls and associated bone fractures compared with their general population counterparts of similar age. Although the cause of falls in patients with PD may be multifactorial, an often under-recognized factor is neurogenic orthostatic hypotension (nOH). nOH is a sustained decrease in blood pressure upon standing whose symptomology can include dizziness/lightheadedness, weakness, fatigue, and syncope. nOH is due to dysfunction of the autonomic nervous system compensatory response to standing and is a consequence of the neurodegenerative processes of PD. The symptoms associated with orthostatic hypotension (OH)/nOH can increase the risk of falls, and healthcare professionals may not be aware of the real-world clinical effect of nOH, the need for routine screening, or the value of early diagnosis of nOH when treating elderly patients with PD. nOH is easily missed and, importantly, healthcare providers may not realize that there are effective treatments for nOH symptoms that could help lessen the fall risk resulting from the condition. This review discusses the burden of, and key risk factors for, falls among patients with PD, with a focus on practical approaches for the recognition, assessment, and successful management of OH/nOH. In addition, insights are provided as to how fall patterns can suggest fall etiology, thereby influencing the choice of intervention.

Parkinson disease (PD) is a chronic and often progressive neurodegenerative disorder that, in more advanced stages, is often associated with postural instability and gait dysfunction. These problems impart an increased risk for falls [1]. The major risk factor for PD is increasing age, and worldwide prevalence estimates range from 0.43% for individuals aged 60 to 69 years, 1% for those 70 to 79 years, and 1.9% for those >80 years [2]. In an aging population with increased survival, the prevalence of PD is expected to increase [3].

The PD population experiences nearly double the risk for falls and associated bone fractures compared with the general population of similar age and health [4]. Consequently, the risk of falls in PD patients represents a major health burden, with a reported annual incidence of 43% to 68% of patients experiencing ≥1 fall [5-11]. Among patients with PD who have already experienced a fall, this problem is recurrent in approximately 30% to 40% of patients [8,12].

One clinical problem associated with falls is orthostatic hypotension (OH), defined as a sustained reduction in blood pressure (BP) within 3 minutes after standing. It can cause symptoms such as dizziness, lightheadedness, and syncope [13]. The link between OH and falls has been established in older persons by several studies [14,15]. In addition, OH has been reported to occur in up to 47% to 58% of PD patients [16-18]. Neurogenic OH (nOH) is a subcategory of OH in which the orthostatic BP reduction results from autonomic failure due to a peripheral or central (or combined) neurological disorder, such as PD, multiple system atrophy, or peripheral autonomic neuropathy [19].

This review discusses the burden of and key risk factors for falls among patients with PD, with a focus on the recognition, assessment, and management of OH/nOH as an important contributing factor.

## Assessment of Orthostatic Hypotension in Parkinson Disease

Because OH can be an important contributor to falls for patients with PD, it is important to recognize the causes and incorporate screening and assessment of postural hypotensive signs and symptoms into the clinical management of PD patients. A recent meta-analysis of 25 studies reported an estimated prevalence of OH in PD patients of 30% [20], emphasizing the need for routine screening.

**Table 1:** Causes of OH and nOH [24-26].

Cause	OH	nOH
Medications	•Dopaminergic agents •Antidepressants (tricyclic antidepressants) •Anticholinergics •Antihypertensives •Diuretics •Nitrates •Phosphodiesterase inhibitors •Vasodilators •Negative inotropic/chronotropic agents •Central sympatholytics •Renin-angiotensin system antagonists	•nOH may be exacerbated by medications that cause OH
Clinical etiologies	•Hypovolemia ◦Dehydration ◦Bleeding •Impaired cardiac output/cardiac pump failure ◦Cardiac arrhythmia ◦Aortic stenosis ◦Heart failure •Venous pooling ◦Prolonged recumbency or standing ◦Postprandial dilation of splanchnic vessel beds ◦Heat exposure ◦Fever	•Primary neurogenic causes ◦Sympathetic noradrenergic denervation ■Parkinson disease ■Pure autonomic failure ◦Intact sympathetic noradrenergic innervation ■Multiple system atrophy ■Dopamine beta-hydroxylase deficiency (intact innervation but norepinephrine deficiency) •Secondary neurogenic causes ◦Peripheral neuropathies ◦Spinal cord problems

nOH=neurogenic orthostatic hypotension; OH=orthostatic hypotension.

### Definition of Orthostatic Hypotension

The American Academy of Neurology and American Autonomic Society consensus statement defines OH as a reduction of systolic BP of ≥20 mmHg or diastolic BP of ≥10 mmHg that occurs within 3 minutes of standing or head-up tilt (HUT) [19,21]. Although this definition focuses on the magnitude of the decline in BP, the actual mean BP while standing may be more clinically relevant to symptomatology; in particular, an actual mean BP <75 mmHg while standing offered high sensitivity and specificity for identifying symptomatic patients [22]. Interestingly, both symptomatic and asymptomatic OH patients reported similar extent of functional impairment in activities of daily living (ADL)/instrumental ADL (iADL) and the Ambulatory Capacity Measure assessments [23]. The implications of the latter observations are that it is important not to limit an analysis to only symptomatic OH patients when evaluating the contribution of OH to disability in patients with PD [23].

### Causes of Orthostatic Hypotension/Neurogenic Orthostatic Hypotension

The origin of OH may be non-neurogenic or neurogenic, and individual patients may have both non-neurogenic and neurogenic causes of OH. It is important to identify the major cause or causes, as this may affect management; common causes are listed in Table 1 [13,24-26]. Among the most common causes of nOH is chronic autonomic failure intrinsic to PD [25,27]. Non-neurogenic causes of OH include the influence of certain medications or clinical conditions that impair cardiac output. Medications used to treat hypertension, depression, or bladder symptoms can induce or exacerbate OH [26]. Anti-hypertensives, in particular, are a common cause of OH [26]. Reduced blood volume associated with inadequate daily fluid intake is also a common factor [24,28]. In addition, levodopa and other dopaminergic therapy for PD can cause hypotensive responses [29]. Based on our clinical experiences, even conventional doses of levodopa pose a risk, especially if an inadequate dose of a peripheral dopamine decarboxylase inhibitor is co-administered (eg, <75 mg/day carbidopa). Drugs that augment levodopa effects (such as monoamine oxidase B and catecholamine-O-methyltransferase inhibitors) or dopaminergic agonists (pramipexole, ropinirole, and rotigotine) also can produce OH [30,31]. Thus, both the presence of PD and its most common treatments can add to the risk for OH.

### Symptoms of Orthostatic Hypotension

The range of symptoms associated with OH is listed in Table 2 [13,24,26,27,32,33]. The most common experiences are dizziness/lightheadedness, presyncope, and syncope, although patients may present with less specific symptoms, such as weakness or fatigue [13]. Symptoms in elderly patients with OH may be more likely to result from age-related factors, such as decreased baroreflex sensitivity, impaired vascular function, and reduced blood flow, resulting in inadequate perfusion of the brain (eg, lightheadedness and syncope) [34]. In an analysis of OH-related hospitalizations in the United States, physiologic changes occurring during aging and risk factors such as neurodegenerative disease were linked to the age-related increase in hospitalizations due to OH [35]. This analysis also reported that PD patients ≥75 years old accounted for 5.2% of OH-related hospitalizations [35].

**Table 2:** Symptoms of OH and nOH [13,24,26,27]*.

Common	•Postural lightheadedness or dizziness •Syncope/presyncope •Visual disturbance •Sensation of blacking out •Falls with or without syncope
Less common	•Orthostatic cognitive dysfunction •Mental dulling •Generalized weakness •Neck pain or discomfort in the suboccipital and paracervical region [“coat hanger” distribution) •Fatigue •Nausea •Headache •Dyspnea

nOH=neurogenic orthostatic hypotension; OH=orthostatic hypotension. *Some patients may be asymptomatic [32,33].

### Screening and Diagnosis of Orthostatic Hypotension

Recent guidelines issued by the American Autonomic Society and the National Parkinson Foundation consensus panel recommend screening patients at increased risk of OH, especially those with suspected or diagnosed neurodegenerative disorder associated with autonomic dysfunction (eg, PD) [26]. The screening process includes inquiries about key symptoms of OH, the frequency and severity of their occurrence, how long the patient is able to stand, and the impact of these symptoms on the patient’s regular activities [26]. Examples of suggested screening questions are included in Table 3 [26]. These recommendations also suggest that specific questions be asked regarding the circumstances of falls, but clinicians are cautioned to consider that patients might not be forthcoming regarding their symptoms or falls for fear of losing their autonomy [26]. Physicians should also consider a number of contributing factors that may cause patient falls, including postural instability, gait impairment, poor vision and proprioception, cognitive impairment, and physical environmental factors [36].

Thorough descriptions of diagnostic strategies to investigate OH have been published [26,37]. An accepted standard for reliable assessment is for a patient to remain in the supine position for ≥5 minutes and then to stand for 3 minutes, with measurement of BP just before standing and at both 1 and 3 minutes of standing. When a supine BP measurement is not feasible, seated-to-standing BP measurements can be a suitable alternative [26]. In a recent study, measuring BP within 1 minute of standing was effective (and, in some ways, a more clinically relevant assessment) [38]. Clinicians can evaluate both the change in BP from supine/seated-to-standing and the actual mean BP at 1 minute of standing as a method of screening for OH [22,26]. A decline of ≥20 mmHg systolic BP or 10 mmHg diastolic BP upon standing is diagnostic for OH, even if asymptomatic; a mean standing BP of ≤75 mmHg could also indicate OH [22,33]. An important correlate of BP measurement is determination of heart rate, which can aid in identifying nOH [33]. An inadequate compensatory increase in heart rate is typical of nOH, whereas an increase >15 bpm is characteristic of transient conditions causing OH (such as dehydration). A recent study assessing orthostatic heart rate changes in individuals with autonomic failure resulting from neurodegenerative synucleinopathies (ie, PD, multiple system atrophy, dementia with Lewy bodies, and pure autonomic failure) reported that patients with nOH experienced twice the drop in systolic BP along with only one-third of the increase in heart rate compared with patients with non-neurogenic OH (both *P*<0.0001) [39]. These results suggest that a ratio of change in heart rate to change in systolic pressure that is <0.5 bpm/mmHg is diagnostic of nOH and could be used to differentiate it from non-neurogenic OH [39]. However, the utility of a heart rate measurement may be diminished in some elderly patients because of the potential for age-related impairment in baroreflex function [33].

The cause of OH can be multifactorial. Even if changes in BP and a lack of compensatory change in heart rate suggest nOH, clinicians should nonetheless consider a concurrent non-neurogenic cause of OH by reviewing medications and other evidence from cardiac and neurological examinations, performing an electro-cardiogram (if appropriate), and conducting biochemical and hematological laboratory testing [26,40]. Specialty testing may be needed to evaluate autonomic reflex arcs and to target a specific diagnosis of underlying autonomic failure. A thorough assessment may include one or more types of autonomic testing, with measurement of plasma catecholamines performed in supine and upright positions in conjunction with cardiovascular autonomic testing, sudomotor function testing (such as the Quantitative Sudomotor Axon Reflex Test or thermoregulatory sweat testing), or ambulatory BP monitoring for detecting episodic OH [26,33,41]. Regarding the assessment of catecholamine levels, it should be noted that these measurements should always be interpreted in the context of other test results because patients with some conditions (eg, multiple system atrophy) may exhibit normal catecholamine plasma levels and severe nOH simultaneously [42].

**Table 3:** Screening Questions for Suspected OH/nOH [26].

Screening Questions*
1.Have you fainted/blacked out recently? 2.Do you feel dizzy or lightheaded upon standing? 3.Do you have vision disturbances when standing? 4.Do you have difficulty breathing when standing? 5.Do you have leg buckling or leg weakness when standing? 6.Do you ever experience neck pain or aching when standing? 7.Do the above symptoms improve or disappear when you sit or lay down? 8.Are the above symptoms worse in the morning or after meals? 9.Have you experienced a fall recently? 10.Are there any other symptoms that you commonly experience when you stand up or within 3-5 minutes of standing up that get better when you sit or lay down?

* Any positive response should prompt further investigation with orthostatic blood pressure measurements. This table has been reproduced from Gibbons, et al. *J Neurol*. 2017; 264:1569 under the Creative Commons Attribution 4.0 International License (https://creativecommons.org/licenses/by/4.0/).

## Burden of parkinson disease-related falls

### Clinical

Falls are associated with increased morbidity in PD patients [8,36]. In a study of PD patients who had fallen in the previous year, approximately one-third of falls resulted in physical injury. Most of these (81%) were soft-tissue injuries [7]. An analysis of 24,831 people aged ≥65 years who were identified through the longitudinal National Long-Term Care Survey found that, among PD patients (n=791), 36% experienced fractures over a period of 5 years [43]. In this survey, hip fractures were reported by 16% of patients in the PD cohort (more than double the odds of a hip fracture in patients without PD) [43]. The increased fracture risk in PD patients was more pronounced for lower-extremity fractures, suggesting that patients with this disorder may be unable to generate a timely reaction to buffer their fall by using their upper extremities. This, in turn, may be associated with a higher rate of injuries to the face, head, neck, trunk, and lower extremities [43]. In addition, PD patients were reported to have more complications after fall-related injuries, including hospitalization [44], which is then associated with higher rates of infection and mortality [45].

### Economic Impact

Both PD and falls in elderly individuals are associated with a significant economic burden. In 2006, the annual healthcare costs for PD patients aged >65 years exceeded costs compared with their counterparts without PD ($21,899 vs $10,732) [46]. Regardless of diagnosis, the average charge for a fall resulting in hospitalization for US patients aged 65 to 74 years was estimated to be more than $24,000 (2008 prices) [47]. In 2010, the PD population incurred $8.1 billion more in medical costs than would be expected for a population without PD, 57% of which can be attributed to increased use of nursing home services [3]. Moreover, recurrent falls can lead to nursing home admission, thereby further increasing costs [36]. Based on recent Medicare estimates, the healthcare expenditure associated with nonfatal falls among adults aged ≥65 years is greater than $30 billion [48].

Given the increased prevalence of fall-related injury among elderly patients with PD, the cost burden associated with falls in this population is likely to be high, although cost data specific to PD-related falls are limited [44,49], and cost data for populations with both PD and OH are even scarcer. In a French study from 1999, mean direct costs per year were significantly higher in patients with parkinsonism and OH compared with patients with parkinsonism but without OH (€737.67/month (€8852/year) vs €512.33/month (€6148/year), *P*<0.05) [50]. Additionally, the cohort with both parkinsonism and OH had substantially greater costs for ancillary care (+87% of mean difference in the OH cohort vs the cohort with PD alone), physician fees (+53%), and private hospitalization (+39%) [50]. A recent US study compared medical costs of PD patients and nOH versus PD without nOH and found that patients with both PD and nOH have significantly higher medical costs for falls ($2260 vs $1049; *P*=0.0002) and total all-cause costs ($31,260 vs $20,910; *P*<0.0001) due to more frequent falls, emergency department visits, and inpatient hospitalizations [51]. Similarly, a retrospective study determined that hospitalization costs were approximately 3 times (+285%; *P*=0.04) higher and overall costs (per patient per year) approximately 2.5 times (+256%; *P*=0.04) higher in patients with PD and OH versus those with PD without OH [52].

**Figure 1.**
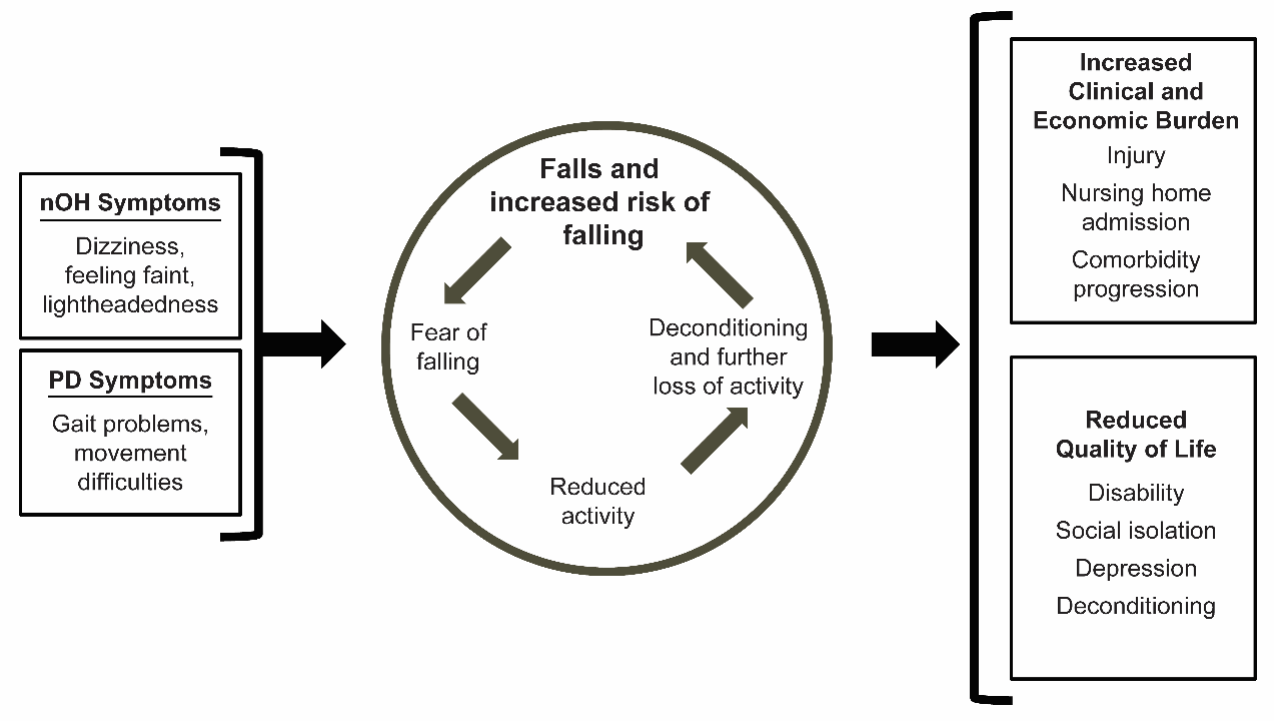
**Impact of falls and fall risk in patients with PD and nOH**. nOH=neurogenic orthostatic hypotension; PD=Parkinson disease [36,55-57].

### Quality of Life Impact

Patient quality of life (QoL) may be affected not only by falls themselves but also by fear of falling. In PD patients, fear of falling has been described as a lack of confidence in the ability to engage in daily activities without falling [53] and is greater in patients who have experienced a fall (even as a rare event) compared with those who have not had this experience [54]. Not surprisingly, fear of falling is a predictor of future falls [55]. Both falls and fear of falling are strongly associated with limitations on activities of daily living and physical inactivity in patients with PD on a crude basis and after adjusting for physical impairments of other categories [54]. Reduced activity due to fear of falling can compound the problems of declining physical health (deconditioning and further loss of activity), which in turn may be factors increasing the risk of falls (Fig. 1) [36,55-57].

The negative QoL effect of falls and associated fear of falling was brought out in a study of elderly patients (n=251) with PD (median duration, 8 years) showing that fear of falling led to avoidance of ordinary ADL, reduced physical activity, and social isolation [58]. Among patients with PD, a history of falls or gait difficulties has been associated with poor or reduced QoL [59,60]. Even when patients have relatively mild PD, a history of one or more previous falls is strongly associated with risk for subsequent falls and fall-related impaired QoL [61]. In addition, OH symptoms have been shown to have a negative impact effect on QoL assessment and ADL of PD patients [62]. Further, PD patients with OH had significantly greater levels of functional impairment compared with PD patients without OH as assessed using the ADL/iADL and the Ambulatory Capacity Measure [23].

### Risk Factors for Falls in Parkinson Disease

In patients with PD, inherent clinical features of PD (especially gait disturbances and postural instability) contribute to the risk of falls. Additionally, patients with PD were shown to have a similar cognitive profile (ie, impaired executive function and attention) to that of elderly individuals (≥65 years old) with a history of unexplained falls, suggesting a role for cognitive impairment in the fall risk of patients with PD [63]. Moreover, a meta-analysis of 6 prospective studies of falls in PD found that the strongest predictor of falls was ≥2 falls during the previous year [64]. In addition, freezing of gait (FOG) has been identified as a strong and independent risk factor for falls [65]. In a study of elderly patients who had ≥1 fall during a 12-month period, more than half (57%) of falls were attributed to intrinsic postural instability (ie, poor balance) or dizziness, followed by accidental causes (eg, stumbling; 39%), and then by syncope (3%) [7]. Finally, cardiovascular autonomic neuropathy has also been shown to be a strong independent predictor of falls, being significantly (odds ratio (OR), 15.194; *P*=0.011) associated with a 15-fold higher probability of falls in patients with PD, whereas patients with PD and OH had a 10-fold higher probability of falls (OR, 10.702; *P*=0.02) [66].

Several risk factors are associated with recurrent falls in PD patients, including longer duration of PD, greater clinical severity, overall functional limitations, more impairment of balance and gait, and levodopa-induced dyskinesia [7,8,65]. An 8-year prospective study found that the percentage of patients reporting falls increased from 41% at baseline to 72% after 8 years [65]. Certain factors associated with falls in patients with PD (such as FOG, gait disturbance, and impaired posture) are associated with increasing disease severity [67]. Motor problems in PD patients, such as FOG, not only increase risk of falls but also are associated with increased risk of fall-related injury [67].

Orthostatic hypotension is an independent risk factor for falls in older individuals [14,15] and is prevalent among patients with PD, with prevalence estimates ranging from 9.6% to 58% [16-18,20]. Patients with PD and OH (whether of neurogenic origin or not) may be more likely to experience a fall than those with PD alone [51,68]. A systematic review and meta-analysis evaluated studies that assessed the association between falls and OH published between 1946 and early 2017 [69]. A subgroup analysis showed that PD patients had the highest odds ratio for the association between falls and OH (OR, 2.30 (95% CI, 1.53-3.48]) compared with the other study populations (ie, community-dwelling adults, geriatric outpatients, geriatric inpatients, nursing home residents, patients with other diseases) [69]. A 2017 study found that 25% of PD + nOH patients experienced a medically attended fall in the past 12 months, whereas 20% of patients with PD alone experienced a fall in the same time frame (*P*=0.016) [51]. In addition, dopaminergic antiparkinsonian medications can contribute to OH or worsen nOH and can be associated with involuntary movements (dyskinesias), both of which have also been linked to increased risk of falls [67]. However, not all studies have found a significant association of OH with increased fall risk in PD. PD patients have several risk factors for falls; therefore, the contribution of OH to fall risk is not easily ascertained from published study data [68]. A prospective cohort study of patients with nOH found that 54% had experienced a fall in the past month [70].

## Assessment of the Risk of Falls: Importance of Different Patterns of Falls

Various assessment tools can be useful to evaluate risk for falls. These include the modified Timed Up and Go test [71], vestibular sensorial organization test, limits of stability of mobile posturography [72], and free-field body sway analysis (such as the VertiGuard device (Vesticure GmbH, Pforzheim, Germany) [72], and all have been useful at determining increased risk for falling in PD patients [71,72]. In addition, a study in PD patients with a history of falls in the previous 6 months showed that the brief-balance evaluation system test reliably predicted risk for future falls [73]. A prospective 12-month study showed that elderly PD patients who were at risk of falls scored higher on a FOG questionnaire compared with age-matched controls [74]. Backward postural instability (retropulsive imbalance), in addition to FOG, is associated with falls in elderly PD patients and can be assessed using the Nutt Retropulsion Test [53]. In a retropulsion test, the patient (standing upright) is pulled backward and the number of corrective steps is counted [75]. Because anticipated retropulsion can yield different results than unanticipated retropulsion, the Nutt Retropulsion Test includes an unexpected shoulder pull [75,76].

Fall patterns may provide insight regarding both etiology and appropriate intervention. For example, falls occurring immediately after rising from sitting or lying positions suggest a strong possibility of either OH or severe balance impairment or both [36]. Patients who report tripping over doorsteps or stairs may benefit from general fall reduction approaches, such as the installation of handrails in the home, use of walking frames, or physical therapy targeted at fall avoidance, such as Lee Silverman Voice Training BIG (LSVT-BIG; www.lsvtglobal.com) [36,77,78].

## Interventions and Treatment for Orthostatic Hypotension and Fall Risk

### Goals of Treatment

The overall goal of treatment is not the normalization of BP but rather to decrease the symptoms of OH [79]. Treatment goals for patients with OH include reducing the risk for falls and fall-associated injuries, prolonging safe standing time, and improving patients’ physical capabilities in terms of mobility and independent functioning [26,80]. Because the use of pressor therapies to treat OH can cause or exacerbate supine hypertension (systolic BP ≥140 mmHg and/or diastolic BP ≥90 mmHg), patients should be monitored to detect if potentially dangerous BP increases occur when in the supine position, and it should be determined whether bedtime antihypertensive therapy intervention is warranted. Such therapy must be balanced against the potential to exacerbate the symptoms of OH [81,82]. Aggressive antihypertensive treatment with the goal of reducing systolic BP below 140 mmHg may not be warranted, particularly in older patients with a history of OH, labile BP, frailty, cognitive impairment, functional limitations, syncope, and falls [83]. In our clinical experience, supine BP below 180/100 mmHg (measured in the patient’s normal sleep position, ideally with the head of the bed elevated by 20-30°) should not be cause for concern and may not necessitate pharmacologic therapy.

### Non-pharmacologic Strategies of Orthostatic Hypotension/Neurogenic Orthostatic Hypotension and Reduction of Fall Risk 

A careful evaluation of current medications may be the first and only intervention needed to control OH [26]. The decision to reduce the dose of or discontinue a medication should be individualized, but particular attention should be paid to any anti-hypertensive medications. Older adults are commonly treated for hypertension, but a dose of an anti-hypertensive medication that was appropriate in an otherwise healthy patient may be too high for a PD patient with attendant autonomic dysfunction because of the increased risk of OH [26]. Patients with PD may also be taking monoamine oxidase B inhibitors, which, in our clinical experience, can lower BP and are only mildly effective for PD symptoms. Clinical experience also suggests that dopamine agonists and amantadine can be considered for discontinuation and that dose reduction of levodopa can even be considered in some cases. A full list of medications whose discontinuation or dose reduction might be of benefit is provided in Table 1 [26]. Shifting the timing of dosing for medications capable of lowering BP or slowing pulse rate may be adequate to address OH symptoms [26,84]. In some patients with OH, avoidance of factors that may induce symptoms (eg, hot environment, carbohydrate-rich meals, and medications capable of lowering BP and not otherwise needed) offers a starting approach to management [26,37].

Examples of non-pharmacologic interventions that can address OH symptoms, such as physical counter-maneuvers and strength training are summarized in Table 4 [24,26,37,77,78,85-87]. Non-pharmacologic inter-ventions that are generally not effective include home-based resistance training and lower body-only compression garments (eg, knee-length compression stockings) [87,88]; in a study of non-pharmacologic interventions, water bolus ingestion, abdominal compression, and physical countermaneuvers were effective in preventing BP drops, while compression stocking did not affect BP drops [89]. Although outcomes from the use of non-pharmacologic interventions for fall prevention in patients with PD and OH have not been extensively studied, some non-pharmacologic interventions are capable of reducing fall risk in PD [77,85,86]. In a study of elderly patients with PD, use of a 4-wheeled walker or the U-Step rollator (Instep Mobility, Skokie, IL) was associated with fewer episodes of freezing, near-falls, and completed falls compared with use of other assistive devices (eg, cane, standard walking frame, or a walking frame with 2 wheels) or no assistive device at all [86]. In another study, vestibular rehabilitation for 8 weeks significantly improved several measures of balance (eg, Berg Balance Scale, Activities-Specific Balance Confidence Scale scores) and gait (Dynamic Gait Index) of elderly patients with PD [85]. In addition, individualized exercises to improve ADL, such as LSVT-BIG, have shown promise in PD patients [77,78]; however, evaluations for PD patients who are also affected with OH have not been performed.

**Table 4:** Non-pharmacologic Interventions That May Reduce OH/nOH Symptoms or General Fall Risk in PD.

Interventions	Address OH/nOH Symptoms	Address General Fall Reduction in PD
Physical counter-maneuvers [37,87,89]	•Leg crossing with active muscle tensing •Bending forward, arms crossed over the abdomen •Squatting •Lower-body muscle tensing after squatting	
Compression garments [26,87,89]	•Abdominal or full-body compression garments	
Other [24,26,89]	•Sleeping with elevation of the head end of the bed (6-9 inches) •Liberal intake of salt, up to 10 g of sodium/day •Adequate hydration (target 2-3 L/day) •Oral water bolus (500 mL)	
Assistive devices/safety measures for general fall reduction [37,86]		•Walking frames •Canes that can be folded into a tripod chair •Handrails
Physical therapy or symptoms of PD that may affect fall risk [77,78,85]		•Vestibular training, Lee Silverman Voice Training BIG (LSVT-BIG)

nOH=neurogenic orthostatic hypotension; OH=orthostatic hypotension; PD=Parkinson disease.

### Pharmacologic Treatment for Orthostatic Hypotension/ Neurogenic Orthostatic Hypotension 

Pharmacologic treatment for OH/nOH is recommended in patients with PD whose symptoms are not relieved by non-pharmacologic approaches [26]. Such treatment should be considered as an initial approach when patients have been experiencing syncope, presyncope, lightheadedness, or falls attributable to a drop in BP [26].

A list of approved and off-label pharmacologic interventions for OH/nOH is provided in Table 5 [26,37,90,91]. Several products (eg, fludrocortisone, indomethacin, pyridostigmine, and dihydroergotamine) are off-label options for the management of OH; however, some are supported only by limited clinical trial data that go back several decades [26,37]. Drugs approved by the US Food and Drug Administration specifically for the treatment of symptomatic OH or nOH include the α_1_-adrenoreceptor prodrug midodrine indicated for OH [90] and the norepinephrine prodrug droxidopa (L-*threo-*dihydroxyphenylserine) indicated for nOH [91]. In Japan, droxidopa (originally known as L-DOPS) has been approved and used since 1989 for OH [92]. In double-blind studies for patients with nOH, treatment with midodrine (10 or 20 mg, 1-3 times daily) resulted in significant improvements in standing systolic and diastolic BP and in symptoms of nOH such as lightheadedness [93,94].

In an integrated analysis of randomized, placebo-controlled, double-blind clinical study data, patients with nOH (two-thirds of whom had an underlying diagnosis of PD) treated with droxidopa exhibited improvements in standing systolic BP compared with placebo. In addition, there was a reduction in symptoms of nOH (such as dizziness and lightheadedness) after 1 week of droxidopa treatment compared with placebo [95]. In another 10-week, multicenter, randomized, double-blind study in 225 patients with PD and symptomatic nOH, droxidopa treatment was associated with fewer falls and fall-related injuries (eg, contusions, lacerations) compared with placebo. In the latter study, the beneficial effects of droxidopa on nOH may have contributed to the observed reduction in falls [96]. Findings of patient experiences in a post hoc analysis of this study suggest that droxidopa also reduced fear of falling [97].

A 6-month, non-interventional, prospective cohort study in 179 patients newly initiating droxidopa for the treatment of nOH (including 59 patients with PD and nOH) showed that significant improvements from baseline were achieved in nOH symptoms (*P*<0.01), functionality (*P*<0.01), and health-related QoL parameters (*P*≤0.002) during 1 month of droxidopa treatment [70]. The improvements in nOH symptoms noted at 1 month persisted at 3 and 6 months during continued treatment [70]. Dizziness/lightheadedness symptoms were improved at all assessments (1, 3, and 6 months; *P*<0.01 for all). The proportion of patients reporting ≥1 fall in the previous month was also reduced from 51% at baseline to 40% at 6 months after starting droxidopa (11% reduction; *P*=0.03) [70]. Other observations from this study at 6 months included significant improvements in fear of falling, functional impairment, depressive symptoms, and health-related QoL scores [70]. However, because this study lacked a contemporaneous control group, the ability to establish causality is limited and additional studies comparing droxidopa with other treatment options in patients with similar comorbidities and disease severity are necessary [70].

In PD patients receiving levodopa and, in some instances, a dopaminergic receptor agonist, the co-administration of droxidopa was associated with improvement in the part 2 ADL scores on the Unified Parkinson’s Disease Rating Scale (UPDRS) in a randomized, placebo-controlled, double-blind study [98]. However, because symptoms of nOH were not evaluated in the patients in this study, it is not possible to determine if the improved UPDRS part 2 scores were related to lessening symptoms of nOH.

**Table 5:** Pharmacologic Treatments for nOH/OH.

Medication	Level of Evidence [99]	Comments
Droxidopa [26,91]	A	•FDA approved for symptomatic nOH
Midodrine [37,90]	A	•FDA approved for symptomatic OH
Fludrocortisone [37]	C	•First-line monotherapy for OH •Full benefit requires high dietary salt and adequate fluid intake
Octreotide [37]	C	•May be used 30 minutes before a meal to reduce postprandial OH
Pyridostigmine [26]	C	•For patients with less severe symptoms with residual sympathetic function
Ephedrine [37]	N/A	•Considered GPP but no clear evidence for use in OH
Yohimbine [37]	N/A	•Considered GPP but no clear evidence for use in OH •Has been used in refractory OH
Dihydroergotamine [37]	N/A	•Considered GPP but no clear evidence for use in OH •Has been used in severe OH
Desmopressin [37]	N/A	•Considered GPP but no clear evidence for use in OH
Erythropoietin [37]	N/A	•Considered GPP but no clear evidence for use in OH •Recommended in anemic patients
Indomethacin [37]	N/A	•Considered GPP but no clear evidence for use in OH •Has been used in severe OH

FDA=US Food and Drug Administration; GPP=good practice point; N/A=not applicable; nOH=neurogenic orthostatic hypotension; OH=orthostatic hypotension.

## Conclusions

The risk of falls and fall-related injuries is high among elderly patients with PD and is associated with a negative clinical impact, reduced QoL, and, consequently, an increased economic burden on healthcare. In patients with PD, OH and nOH are important risk factors for falls and further contribute to the burden of disease. Screening for OH/nOH and consideration of appropriate non-pharmacologic strategies and pharmacologic treatments (including lowering the doses of medications that may exacerbate symptoms) are important in the management of elderly patients with PD. The multifactorial risk profile for falls in patients with PD suggests that clinicians should consider a broad range of etiologies, including commonly used medications for lowering BP, when planning treatment management strategies.

## References

[1] World Health Organization. Neurological disorders: public health challenges. Available at: www.who.int/mental_health/neurology/neurodiso/en/. Accessed August 7, 2019.

[2] Pringsheim T, Jette N, Frolkis A, et al (2014). The prevalence of Parkinson's disease: a systematic review and meta-analysis. Mov Disord, 29(13):1583-90. 24976103 10.1002/mds.25945

[3] Kowal SL, Dall TM, Chakrabarti R, et al (2013). The current and projected economic burden of Parkinson's disease in the United States. Mov Disord, 28(3):311-8. 23436720 10.1002/mds.25292

[4] Kalilani L, Asgharnejad M, Palokangas T, et al (2016). Comparing the incidence of falls/fractures in Parkinson's disease patients in the US population. PLoS One, 11(9):e0161689. 27583564 10.1371/journal.pone.0161689 PMC5008740

[5] Canning CG, Paul SS, Nieuwboer A (2014). Prevention of falls in Parkinson's disease: a review of fall risk factors and the role of physical interventions. Neurodegener Dis Manag, 4(3):203-21. 25095816 10.2217/nmt.14.22

[6] Wood BH, Bilclough JA, Bowron A, et al (2002). Incidence and prediction of falls in Parkinson's disease: a prospective multidisciplinary study. J Neurol Neurosurg Psychiatry, 72(6):721-5. 12023412 10.1136/jnnp.72.6.721 PMC1737913

[7] Allcock LM, Rowan EN, Steen IN, et al (2009). Impaired attention predicts falling in Parkinson's disease. Parkinsonism Relat Disord, 15(2):110-5. 18487069 10.1016/j.parkreldis.2008.03.010

[8] Almeida LR, Valenca GT, Negreiros NN, et al (2014). Recurrent falls in people with Parkinson's disease without cognitive impairment: focusing on modifiable risk factors. Parkinsons Dis, 2014:432924. 25506466 10.1155/2014/432924 PMC4259076

[9] Ashburn A, Stack E, Pickering RM, et al (2001). A community-dwelling sample of people with Parkinson's disease: characteristics of fallers and non-fallers. Age Ageing, 30(1):47-52. 11322672 10.1093/ageing/30.1.47

[10] Latt MD, Lord SR, Morris JG, et al (2009). Clinical and physiological assessments for elucidating falls risk in Parkinson's disease. Mov Disord, 24(9):1280-9. 19425059 10.1002/mds.22561

[11] Paul SS, Canning CG, Sherrington C, et al (2013). Three simple clinical tests to accurately predict falls in people with Parkinson's disease. Mov Disord, 28(5):655-62. 23450694 10.1002/mds.25404

[12] Rudzinska M, Bukowczan S, Stozek J, et al (2013). Causes and consequences of falls in Parkinson disease patients in a prospective study. Neurol Neurochir Pol, 47(5):423-30. 24166563 10.5114/ninp.2013.38222

[13] Freeman R, Wieling W, Axelrod FB, et al (2011). Consensus statement on the definition of orthostatic hypotension, neurally mediated syncope and the postural tachycardia syndrome. Clin Auton Res, 21(2):69-72. 21431947 10.1007/s10286-011-0119-5

[14] McDonald C, Pearce M, Kerr SR, et al (2017). A prospective study of the association between orthostatic hypotension and falls: definition matters. Age Ageing, 46(3):439-45. 28013227 10.1093/ageing/afw227

[15] Ooi WL, Hossain M, Lipsitz LA (2000). The association between orthostatic hypotension and recurrent falls in nursing home residents. Am J Med, 108(2):106-11. 11126303 10.1016/s0002-9343(99)00425-8

[16] Allcock LM, Ullyart K, Kenny RA, et al (2004). Frequency of orthostatic hypotension in a community based cohort of patients with Parkinson's disease. J Neurol Neurosurg Psychiatry, 75(10):1470-1. 15377699 10.1136/jnnp.2003.029413 PMC1738761

[17] Senard JM, Rai S, Lapeyre-Mestre M, et al (1997). Prevalence of orthostatic hypotension in Parkinson's disease. J Neurol Neurosurg Psychiatry, 63(5):584-9. 9408097 10.1136/jnnp.63.5.584 PMC2169808

[18] Fereshtehnejad SM, Lokk J (2014). Orthostatic hypotension in patients with Parkinson's disease and atypical parkinsonism. Parkinsons Dis, 2014:475854. 10.1155/2014/475854 PMC3929346 24634790

[19] Freeman R, Abuzinadah AR, Gibbons C, et al (2018). Orthostatic hypotension: JACC state-of-the-art review. J Am Coll Cardiol, 72(11):1294-309. 30190008 10.1016/j.jacc.2018.05.079

[20] Velseboer DC, de Haan RJ, Wieling W, et al (2011). Prevalence of orthostatic hypotension in Parkinson's disease: a systematic review and meta-analysis. Parkinsonism Relat Disord, 17(10):724-9. 21571570 10.1016/j.parkreldis.2011.04.016 PMC5199613

[21] Consensus Committee of the American Autonomic Society and the American Academy of Neurology (1996). Consensus statement on the definition of orthostatic hypotension, pure autonomic failure, and multiple system atrophy. The Consensus Committee of the American Autonomic Society and the American Academy of Neurology. Neurology, 46(5):1470. 8628505 10.1212/wnl.46.5.1470

[22] Palma JA, Gomez-Esteban JC, Norcliffe-Kaufmann L, et al (2015). Orthostatic hypotension in Parkinson disease: how much you fall or how low you go? Mov Disord, 30(5):639-45. 25678194 10.1002/mds.26079 PMC4397106

[23] Merola A, Romagnolo A, Rosso M, et al (2016). Orthostatic hypotension in Parkinson's disease: does it matter if asymptomatic? Parkinsonism Relat Disord, 33:65-71. 27641792 10.1016/j.parkreldis.2016.09.013

[24] Jones PK, Shaw BH, Raj SR (2015). Orthostatic hypotension: managing a difficult problem. Expert Rev Cardiovasc Ther, 13(11):1263-76. 26427904 10.1586/14779072.2015.1095090 PMC4883667

[25] Goldstein DS, Sharabi Y (2009). Neurogenic orthostatic hypotension: a pathophysiological approach. Circulation, 119(1):139-46. 19124673 10.1161/CIRCULATIONAHA.108.805887 PMC4182314

[26] Gibbons CH, Schmidt P, Biaggioni I, et al (2017). The recommendations of a consensus panel for the screening, diagnosis, and treatment of neurogenic orthostatic hypotension and associated supine hypertension. J Neurol, 264(8):1567-82. 28050656 10.1007/s00415-016-8375-x PMC5533816

[27] Kuritzky L, Espay AJ, Gelblum J, et al (2015). Diagnosing and treating neurogenic orthostatic hypotension in primary care. Postgrad Med, 127(7):702-15. 26012731 10.1080/00325481.2015.1050340

[28] Weinberg AD, Minaker KL (1995). Dehydration. Evaluation and management in older adults. Council on Scientific Affairs, American Medical Association. JAMA, 274(19):1552-6. 7474224 10.1001/jama.274.19.1552

[29] Calne DB, Brennan J, Spiers AS, et al (1970). Hypotension caused by L-dopa. Br Med J, 1(5694):474-5. 4907812 10.1136/bmj.1.5694.474 PMC1699425

[30] Jankovic J, Stacy M (2007). Medical management of levodopa-associated motor complications in patients with Parkinson's disease. CNS Drugs, 21(8):677-92. 17630819 10.2165/00023210-200721080-00005

[31] Dewey RB Jr., (2004). Management of motor complications in Parkinson's disease. Neurology, 62(6 suppl 4):S3-7. 10.1212/wnl.62.6_suppl_4.s3 15037664

[32] Sclater A, Alagiakrishnan K (2004). Orthostatic hypotension. A primary care primer for assessment and treatment. Geriatrics, 59(8):22-7. 15332413

[33] Shibao C, Lipsitz LA, Biaggioni I (2013). ASH position paper: evaluation and treatment of orthostatic hypotension. J Clin Hypertens (Greenwich), 15(3):147-53. 23458585 10.1111/jch.12062 PMC8033893

[34] Maule S, Papotti G, Naso D, et al (2007). Orthostatic hypotension: evaluation and treatment. Cardiovasc Hematol Disord Drug Targets, 7(1):63-70. 17346129 10.2174/187152907780059029

[35] Shibao C, Grijalva CG, Raj SR, et al (2007). Orthostatic hypotension-related hospitalizations in the United States. Am J Med, 120(11):975-80. 17976425 10.1016/j.amjmed.2007.05.009

[36] Voermans NC, Snijders AH, Schoon Y, et al (2007). Why old people fall (and how to stop them). Pract Neurol, 7(3):158-71. 17515595 10.1136/jnnp.2007.120980

[37] Lahrmann H, Cortelli P, Hilz M, et al (2006). EFNS guidelines on the diagnosis and management of orthostatic hypotension. Eur J Neurol, 13(9):930-6. 16930356 10.1111/j.1468-1331.2006.01512.x

[38] Juraschek SP, Daya N, Rawlings AM, et al (2017). Association of history of dizziness and long-term adverse outcomes with early vs later orthostatic hypotension assessment times in middle-aged adults. JAMA Intern Med, 177(9):1316-23. 28738139 10.1001/jamainternmed.2017.2937 PMC5661881

[39] Norcliffe-Kaufmann L, Kaufmann H, Palma JA, et al (2018). Orthostatic heart rate changes in patients with autonomic failure caused by neurodegenerative synucleinopathies. Ann Neurol, 83(3):522-31. 29405350 10.1002/ana.25170 PMC5867255

[40] Freeman R (2008). Clinical practice. Neurogenic orthostatic hypotension. N Engl J Med, 358(6):615-24. 18256396 10.1056/NEJMcp074189

[41] Milazzo V, Di Stefano C, Vallelonga F, et al (2018). Reverse blood pressure dipping as marker of dysautonomia in Parkinson disease. Parkinsonism Relat Disord, 56:82-7. 30057156 10.1016/j.parkreldis.2018.06.032

[42] Goldstein DS, Holmes C, Sharabi Y, et al (2003). Plasma levels of catechols and metanephrines in neurogenic orthostatic hypotension. Neurology, 60(8):1327-32. 12707437 10.1212/01.wnl.0000058766.46428.f3

[43] Pressley JC, Louis ED, Tang MX, et al (2003). The impact of comorbid disease and injuries on resource use and expenditures in parkinsonism. Neurology, 60(1):87-93. 12525724 10.1212/wnl.60.1.87

[44] Paul SS, Harvey L, Canning CG, et al (2017). Fall-related hospitalization in people with Parkinson's disease. Eur J Neurol, 24(3):523-9. 28117538 10.1111/ene.13238

[45] Huang YF, Cherng YG, Hsu SP, et al (2015). Risk and adverse outcomes of fractures in patients with Parkinson's disease: two nationwide studies. Osteoporos Int, 26(6):1723-32. 25672807 10.1007/s00198-015-3052-y

[46] Noyes K, Liu H, Li Y, et al (2006). Economic burden associated with Parkinson's disease on elderly Medicare beneficiaries. Mov Disord, 21(3):362-72. 16211621 10.1002/mds.20727

[47] Davis JC, Robertson MC, Ashe MC, et al (2010). International comparison of cost of falls in older adults living in the community: a systematic review. Osteoporos Int, 21(8):1295-306. 20195846 10.1007/s00198-009-1162-0

[48] Burns ER, Stevens JA, Lee R (2016). The direct costs of fatal and non-fatal falls among older adults - United States. J Safety Res, 58:99-103. 27620939 10.1016/j.jsr.2016.05.001 PMC6823838

[49] Wielinski CL, Erickson-Davis C, Wichmann R, et al (2005). Falls and injuries resulting from falls among patients with Parkinson's disease and other parkinsonian syndromes. Mov Disord, 20(4):410-5. 15580552 10.1002/mds.20347

[50] Desboeuf K, Grau M, Riche F, et al (2006). Prevalence and costs of parkinsonian syndromes associated with orthostatic hypotension. Therapie, 61(2):93-9. 16886700 10.2515/therapie:2006020

[51] Francois C, Biaggioni I, Shibao C, et al (2017). Fall-related healthcare use and costs in neurogenic orthostatic hypotension with Parkinson's disease. J Med Econ, 20(5):525-32. 28125950 10.1080/13696998.2017.1284668

[52] Merola A, Sawyer RP, Artusi CA, et al (2017). Orthostatic hypotension in Parkinson disease: impact on health care utilization. Parkinsonism Relat Disord, 47:45-9. 29195729 10.1016/j.parkreldis.2017.11.344

[53] Lindholm B, Hagell P, Hansson O, et al (2014). Factors associated with fear of falling in people with Parkinson's disease. BMC Neurol, 14:19. 24456482 10.1186/1471-2377-14-19 PMC3904169

[54] Bryant MS, Rintala DH, Hou JG, et al (2015). Relationship of falls and fear of falling to activity limitations and physical inactivity in Parkinson's disease. J Aging Phys Act, 23(2):187-93. 24700259 10.1123/japa.2013-0244

[55] Mak MK, Pang MY (2009). Fear of falling is independently associated with recurrent falls in patients with Parkinson's disease: a 1-year prospective study. J Neurol, 256(10):1689-95. 19479166 10.1007/s00415-009-5184-5

[56] Allen NE, Schwarzel AK, Canning CG (2013). Recurrent falls in Parkinson's disease: a systematic review. Parkinsons Dis, 2013:906274. 10.1155/2013/906274 PMC3606768 23533953

[57] Bloem BR, van Vugt JP, Beckley DJ (2001). Postural instability and falls in Parkinson's disease. Adv Neurol, 87:209-23. 11347224

[58] Kader M, Iwarsson S, Odin P, et al (2016). Fall-related activity avoidance in relation to a history of falls or near falls, fear of falling and disease severity in people with Parkinson's disease. BMC Neurol, 16:84. 27250988 10.1186/s12883-016-0612-5 PMC4890527

[59] Rascol O, Perez-Lloret S, Damier P, et al (2015). Falls in ambulatory non-demented patients with Parkinson's disease. J Neural Transm, 122:1447-55. 25845678 10.1007/s00702-015-1396-2

[60] Schrag A, Jahanshahi M, Quinn N (2000). What contributes to quality of life in patients with Parkinson's disease? J Neurol Neurosurg Psychiatry, 69(3):308-12. 10945804 10.1136/jnnp.69.3.308 PMC1737100

[61] Voss TS, Elm JJ, Wielinski CL, et al (2012). Fall frequency and risk assessment in early Parkinson's disease. Parkinsonism Relat Disord, 18(7):837-41. 22542094 10.1016/j.parkreldis.2012.04.004 PMC3424355

[62] Magerkurth C, Schnitzer R, Braune S (2005). Symptoms of autonomic failure in Parkinson's disease: prevalence and impact on daily life. Clin Auton Res, 15(2):76-82. 15834763 10.1007/s10286-005-0253-z

[63] Hausdorff JM, Doniger GM, Springer S, et al (2006). A common cognitive profile in elderly fallers and in patients with Parkinson's disease: the prominence of impaired executive function and attention. Exp Aging Res, 32(4):411-29. 16982571 10.1080/03610730600875817 PMC1868891

[64] Pickering RM, Grimbergen YA, Rigney U, et al (2007). A meta-analysis of six prospective studies of falling in Parkinson's disease. Mov Disord, 22(13):1892-1900. 17588236 10.1002/mds.21598

[65] Hiorth YH, Larsen JP, Lode K, et al (2014). Natural history of falls in a population-based cohort of patients with Parkinson's disease: an 8-year prospective study. Parkinsonism Relat Disord, 20(10):1059-64. 25048614 10.1016/j.parkreldis.2014.06.023

[66] Romagnolo A, Zibetti M, Merola A, et al (2019). Cardiovascular autonomic neuropathy and falls in Parkinson disease: a prospective cohort study. J Neurol, 266(1):85-91. 30382389 10.1007/s00415-018-9104-4

[67] Gray P, Hildebrand K (2000). Fall risk factors in Parkinson's disease. J Neurosci Nurs, 32(4):222-8. 10994536 10.1097/01376517-200008000-00006

[68] Matinolli M, Korpelainen JT, Korpelainen R, et al (2009). Orthostatic hypotension, balance and falls in Parkinson's disease. Mov Disord, 24(5):745-51. 19133666 10.1002/mds.22457

[69] Mol A, Bui Hoang PTS, Sharmin S, et al (2019). Orthostatic hypotension and falls in older adults: a systematic review and meta-analysis. J Am Med Dir A, 20(5):589-597 10.1016/j.jamda.2018.11.003 30583909

[70] François C, Shibao CA, Biaggioni I, et al (2019). Six-month use of droxidopa for neurogenic orthostatic hypotension. Mov Disord Clin Pract, 6(3):235-42. 30949555 10.1002/mdc3.12726 PMC6417751

[71] Alexandre TS, Meira DM, Rico NC, et al (2012). Accuracy of Timed Up and Go Test for screening risk of falls among community-dwelling elderly. Rev Bras Fisioter, 16(5):381-8. 10.1590/s1413-35552012005000041 22858735

[72] Rossi-Izquierdo M, Basta D, Rubio-Rodriguez JP, et al (2014). Is posturography able to identify fallers in patients with Parkinson's disease? Gait Posture, 40(1):53-7. 24629311 10.1016/j.gaitpost.2014.02.003

[73] Duncan RP, Leddy AL, Cavanaugh JT, et al (2013). Comparative utility of the BESTest, mini-BESTest, and brief-BESTest for predicting falls in individuals with Parkinson disease: a cohort study. Phys Ther, 93(4):542-50. 23174567 10.2522/ptj.20120302 PMC3613340

[74] Rudzinska M, Bukowczan S, Stozek J, et al (2013). The incidence and risk factors of falls in Parkinson disease: prospective study. Neurol Neurochir Pol, 47(5):431-7. 24166564 10.5114/ninp.2013.38223

[75] Nonnekes J, Goselink R, Weerdesteyn V, et al (2015). The retropulsion test: a good evaluation of postural instability in Parkinson's disease? J Parkinsons Dis, 5(1):43-7. 25613349 10.3233/JPD-140514

[76] Lindholm B, Hagell P, Hansson O, et al (2015). Prediction of falls and/or near falls in people with mild Parkinson's disease. PLoS One, 10(1):e0117018. 25635687 10.1371/journal.pone.0117018 PMC4311993

[77] Janssens J, Malfroid K, Nyffeler T, et al (2014). Application of LSVT BIG intervention to address gait, balance, bed mobility, and dexterity in people with Parkinson disease: a case series. Phys Ther, 94(7):1014-23. 24557655 10.2522/ptj.20130232

[78] Ebersbach G, Grust U, Ebersbach A, et al (2015). Amplitude-oriented exercise in Parkinson's disease: a randomized study comparing LSVT-BIG and a short training protocol. J Neural Transm (Vienna), 122(2):253-6. 24872078 10.1007/s00702-014-1245-8

[79] Palma JA, Kaufmann H (2017). Epidemiology, diagnosis, and management of neurogenic orthostatic hypotension. Mov Disord Clin Pract, 4(3):298-308. 28713844 10.1002/mdc3.12478 PMC5506688

[80] Arbique D, Cheek D, Welliver M, et al (2014). Management of neurogenic orthostatic hypotension. J Am Med Dir Assoc, 15(4):234-9. 24388946 10.1016/j.jamda.2013.10.014

[81] Jordan J, Fanciulli A, Tank J, et al (2019). Management of supine hypertension in patients with neurogenic orthostatic hypotension: scientific statement of the American Autonomic Society, European Federation of Autonomic Societies, and the European Society of Hypertension. J Hypertens, 37(8):1541-6. 30882602 10.1097/HJH.0000000000002078

[82] Espay AJ, LeWitt PA, Hauser RA, et al (2016). Neurogenic orthostatic hypotension and supine hypertension in Parkinson's disease and related synucleinopathies: prioritisation of treatment targets. Lancet Neurol, 15(9):954-66. 27478953 10.1016/S1474-4422(16)30079-5

[83] Scott IA, Hilmer SN, Le Couteur DG (2019). Going Beyond the Guidelines in Individualising the Use of Antihypertensive Drugs in Older Patients. Drugs Aging, 36(8):675-85. 31175614 10.1007/s40266-019-00683-8

[84] Hohler AD, Amariei DE, Katz DI, et al (2012). Treating orthostatic hypotension in patients with Parkinson's disease and atypical parkinsonism improves function. J Parkinsons Dis, 2(3):235-40. 23938231 10.3233/JPD-2012-012101

[85] Acarer A, Karapolat H, Celebisoy N, et al (2015). Is customized vestibular rehabilitation effective in patients with Parkinson's? NeuroRehabilitation, 37(2):255-62. 26484517 10.3233/NRE-151258

[86] Kegelmeyer DA, Parthasarathy S, Kostyk SK, et al (2013). Assistive devices alter gait patterns in Parkinson disease: advantages of the four-wheeled walker. Gait Posture, 38(1):20-4. 23237981 10.1016/j.gaitpost.2012.10.027

[87] Mills PB, Fung CK, Travlos A, et al (2015). Nonpharmacologic management of orthostatic hypotension: a systematic review. Arch Phys Med Rehabil, 96(2):366-75. 25449193 10.1016/j.apmr.2014.09.028

[88] Smeenk HE, Koster MJ, Faaij RA, et al (2014). Compression therapy in patients with orthostatic hypotension: a systematic review. Neth J Med, 72(2):80-5. 24659590

[89] Newton JL, Frith J (2018). The efficacy of nonpharmacologic intervention for orthostatic hypotension associated with aging. Neurology, 91(7):e652-e6. 30006412 10.1212/WNL.0000000000005994 PMC6105042

[90] ProAmatine^®^ (midodrine hydrochloride). Full Prescribing Information, Shire US Inc, Lexington, MA, 2017.

[91] NORTHERA^®^ (droxidopa). Full Prescribing Information, Lundbeck NA Ltd, Deerfield, IL, 2017.

[92] Goldstein DS (2006). L-dihydroxyphenylserine (L-DOPS): a norepinephrine prodrug. Cardiovasc Drug Rev, 24(3-4):189-203. 17214596 10.1111/j.1527-3466.2006.00189.x

[93] Wright RA, Kaufmann HC, Perera R, et al (1998). A double-blind, dose-response study of midodrine in neurogenic orthostatic hypotension. Neurology, 51(1):120-4. 9674789 10.1212/wnl.51.1.120

[94] Low PA, Gilden JL, Freeman R, et al (1997). Efficacy of midodrine vs placebo in neurogenic orthostatic hypotension. A randomized, double-blind multicenter study. Midodrine Study Group. JAMA, 277(13):1046-51. 9091692

[95] Biaggioni I, Arthur Hewitt L, Rowse GJ, et al (2017). Integrated analysis of droxidopa trials for neurogenic orthostatic hypotension. BMC Neurol, 17(1):90. 28494751 10.1186/s12883-017-0867-5 PMC5427571

[96] Hauser RA, Heritier S, Rowse GJ, et al (2016). Droxidopa and reduced falls in a trial of Parkinson patients with neurogenic orthostatic hypotension. Clin Neuropharmacol, 39(5):220-6. 27332626 10.1097/WNF.0000000000000168 PMC5028156

[97] Isaacson S, Francois C, Peng G, et al Effect of droxidopa on fear of falling. Presented at: 19th International Congress of Parkinson’s Disease and Movement Disorders, June 14-18, 2015; San Diego, CA.

[98] Zhao S, Cheng R, Zheng J, et al (2015). A randomized, double-blind, controlled trial of add-on therapy in moderate-to-severe Parkinson's disease. Parkinsonism Relat Disord, 21(10):1214-8. 26342560 10.1016/j.parkreldis.2015.08.023

[99] John Wiley & Sons. Levels of evidence. Available at: https://www.essentialevidenceplus.com/product/ebm_loe.cfm?show=grade. Accessed August 7, 2019.

